# DUSP4 promotes doxorubicin resistance in gastric cancer through epithelial-mesenchymal transition

**DOI:** 10.18632/oncotarget.21522

**Published:** 2017-10-04

**Authors:** Xing Kang, Minhuan Li, Hao Zhu, Xiaofeng Lu, Ji Miao, Shangce Du, Xuefeng Xia, Wenxian Guan

**Affiliations:** ^1^ Department of General Surgery, The Affiliated Drum Tower Hospital of Nanjing University Medical School, Nanjing 210008, Jiangsu Province, China; ^2^ Department of Laboratory Medicine, Sir Run Run Hospital, Nanjing Medical University, Nanjing, 210000, Jiangsu Province, China; ^3^ Department of Gastroenterology, The Afflicted Drum Tower Hospital, Nanjing University Medical School, Nanjing 210008, Jiangsu Province, China

**Keywords:** chemoresistance, doxorubicin, DUSP4, epithelial-mesenchymal transition, gastric cancer

## Abstract

Chemoresistance limits treatment efficacy in gastric cancer and doxorubicin resistance is common in gastric cancer cells. Dual specificity phosphatase 4 (DUSP4) has been associated with tumor progression. This study aimed to investigate the mechanism of DUSP4 regulating doxorubicin resistance in gastric cancer cells. Cell Counting Kit-8 (CCK-8) and 5-ethynyl-2′-deoxyuridine (EdU) incorporation assay were used to measure cell viability and proliferation in gastric cancer cells treated with doxorubicin. The expression of DUSP4, E-cadherin and Vimentin protein was detected by Western blotting. Overexpression of DUSP4 was more resistant to doxorubicin in gastric cancer cells. Knockdown of DUSP4 increased the sensitivity of gastric cancer cells to doxorubicin. Moreover, up-regulation of DUSP4 promoted the Epithelial-Mesenchymal Transition (EMT) in gastric cancer cells, but blocking the EMT using a Twist siRNA increased the sensitivity of gastric cancer cells to doxorubicin and confirmed the EMT was involved in DUSP4-mediated doxorubicin resistance. These findings demonstrated that DUSP4 could enhance doxorubicin resistance by promoting EMT in gastric cancer cells.

## INTRODUCTION

Gastric cancer (GC) is one of the most common digestive system malignancies and has a high mortality rate, accounting for approximately 9% of cancer deaths worldwide [1]. Most patients with newly diagnosed GC have advanced stage disease that is not resectable [2, 3]. Moreover, patients who undergo complete tumor resection have a high risk of recurrence [4]. Chemotherapy has been widely adopted to prolong survival in patients with advanced GC. However, the overall survival rate of advanced GC remains unsatisfactory due to the development of multidrug resistance [5, 6]. Therefore, the mechanisms underlying chemotherapy resistance in GC are of major interest.

Doxorubicin (DOX) is an anthracycline-based chemotherapeutic agent that has been used as the gold-standard therapy for advanced GC since 1980 [7]. However, DOX-based regimens are not currently recommended as a first-line chemotherapy strategy for GC due to frequent development of resistance and poor efficacy. The epithelial-to-mesenchymal transition (EMT) is a major mechanism associated with drug resistance. The EMT has been reported to promote cancer cell metastasis and resistance to chemotherapy [8, 9]. Recent studies demonstrated that the frequent acquisition of DOX resistance in cancer may be related to the EMT [10–14]. The protein alterations that occur during the EMT include a loss of epithelial markers, such as E-cadherin, and acquisition of mesenchymal markers, such as vimentin [15]. Several studies have investigated the relationship between the EMT and DOX-resistance in GC, and many factors involved in EMT mediated DOX-resistance have been discovered [16, 17]. However, the precise mechanisms by which the EMT is associated with DOX-resistance have not yet been fully uncovered.

Dual specificity phosphatase 4 (DUSP4), a member of the DUSP family, is also known as MAPK phosphatase 2 (MKP2). DUSP4 plays an essential role in regulation of cell proliferation and differentiation via interacting with the MAPK signaling pathway [18]. However, contradictory roles have been reported for DUSP4 in cancer. As a negative regulator of the MAPK signaling pathway, DUSP4 may represent as a tumor suppressor gene. Downregulation of DUSP4 is associated with progression in several types of cancer, including colorectal cancer [19], breast cancer [20], pancreatic cancer [21] and diffuse large B cell lymphoma [22]. However, other studies indicate that overexpression of DUSP4 promotes cancer development and progression [23–25]. Interestingly, DUSP4 has been related to drug resistance in several cancers [26–30]. However, little is known about the role of DUSP4 in DOX resistance in GC.

In the present study, we aimed to explore the role of DUSP4 in DOX resistance in GC cell lines, uncover the associated mechanisms, and investigate the link between the EMT and doxorubicin resistance in gastric cancer.

## RESULTS

### GC cells expressing low levels of DUSP4 are more sensitive to DOX

**Figure 1:**
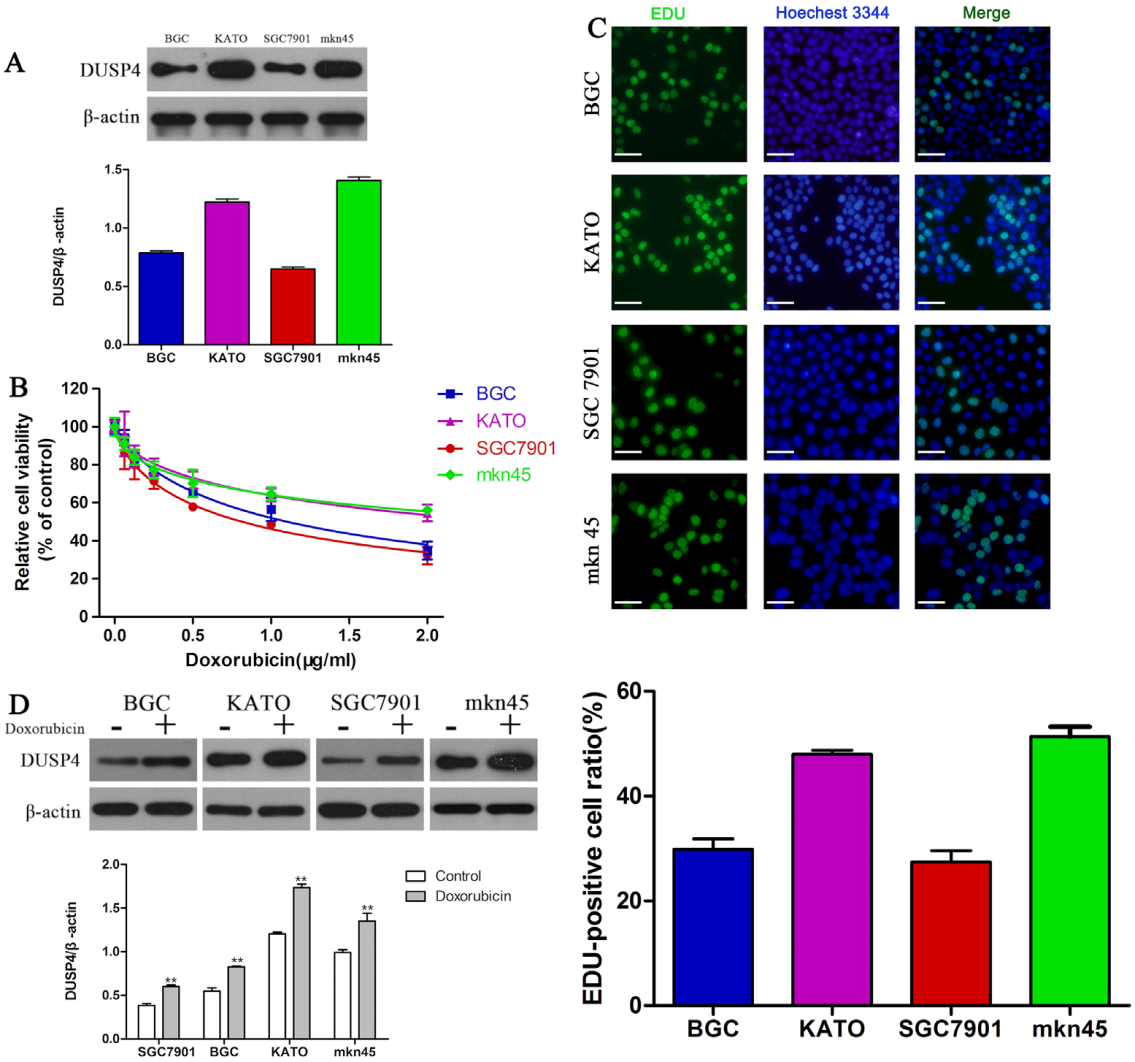
Expression of DUSP4 is associated with DOX resistance in GC cells **(A)** Western blot of DUSP4 protein expression in GC cells. **(B)** CCK-8 assay of the viability of GC cells treated with different concentrations of DOX. **(C)** EDU assay of the proliferation of GC cells treated with their IC_50_ of DOX. **(D)** Western blot of DUSP4 protein expression in GC cells cultured with or without their IC_50_ of DOX. (^**^P < 0.01).

To explore the effect of DUSP4 on the sensitivity of GC cells to DOX, the expression of DUSP4 was measured by Western blotting. KATOIII and MKN45 cells expressed high levels of DUSP4 compared to BGC and SGC7901 cells (Figure 1A). Next, we measured cell viability after 48 h DOX treatment using the CCK-8 assay. All four GC cells exhibited dose-dependent reductions in cell viability in response to DOX (Figure 1B, Table 1). However, DUSP4^high^ KATO III and MKN45 cells were more resistant to DOX than DUSP4^low^ BGC and SGC7901 cells. The EDU cell proliferation assay confirmed these results (Figure 1C). The IC_50_ values of DOX in KATOIII, MKN45, BGC and SGC7901 cells at 48 h were 2.507, 3.025, 1.103 and 0.8146 μg/mL, respectively (Table 1). Moreover, treatment with DOX at the IC_50_ values for 48 h obviously increased the expression of DUSP4 in all four GC cell lines (Figure 1D) (^**^P < 0.01). Taken together, we deduced that DUSP4 may mediate DOX resistance in GC cells.

**Table 1:** The viability of GC cells treated with different concentrations of DOX

cell lines	SGC7901	BGC	KATO	mkn45
IC_50_(μg/ml)	0.8146(0.7179 to 0.9112)	1.103(0.9786 to 1.227)	2.507(1.760 to 3.254)	3.025(2.205 to 3.845)

IC_50_ values show Doxorubicin concentration [μg/ml. mean (95% confidence intervals)].

### DUSP4 promotes DOX resistance in GC cells

**Figure 2:**
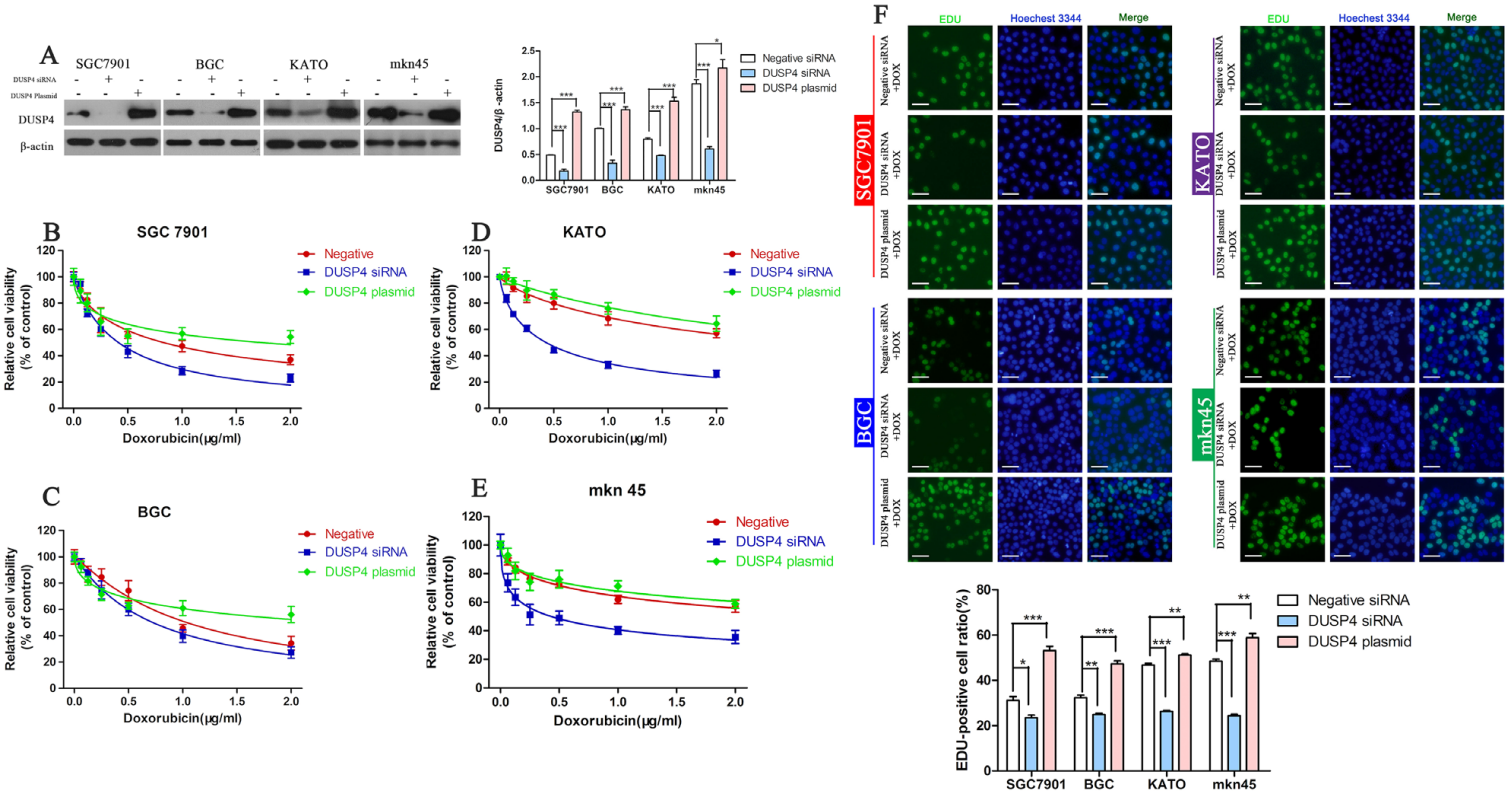
Knockdown of *DUSP4* increases the sensitivity of GC cells to DOX **(A)** Western blot confirmation of the efficiency of DUSP4 knockdown or overexpression; β-actin was used as the internal control (^*^P < 0.05, ^***^P < 0.001). **(B-E)** CCK-8 assay of the viability of GC cells in which DUSP4 was knocked down or overexpressed treated with different concentrations of DOX. **(F)** EDU assay of the proliferation of GC cells in which DUSP4 was knocked down or overexpressed treated with their IC_50_ of DOX. (^*^P < 0.05, ^**^P < 0.01, ^***^P < 0.001).

To further explore the relationship between DUSP4 and DOX resistance in GC cells, we employed a siRNA to knockdown *DUSP4* and a plasmid to overexpress DUSP4. The efficacy of the siRNA and plasmid were confirmed by Western blotting (Figure 2A) (^*^P < 0.05, ^***^P < 0.001). The CCK-8 assay revealed knockdown of *DUSP4* significantly enhanced the cytotoxicity of DOX in GC cells. Conversely, overexpression of DUSP4 significantly reduced the cytotoxicity of DOX (Figure 2B-2E, Table 2). The EDU cell proliferation assay confirmed the results of the CCK-8 assay (Figure 2F) (^*^P < 0.05, ^**^P < 0.01,^***^P < 0.001). These data confirmed that DUSP4 significantly enhances the resistance of GC cells to DOX.

**Table 2:** The viability of GC cells in which DUSP4 was knocked down or overexpressed treated with different concentrations of DOX

cell lines	IC_50_(μg/ml)		
	Negative+ DOX	DUSP4 siRNA+ DOX	DUSP4 plasmid+DOX
SGC7901	0.831(0.7142 to 0.9478)	0.4135(0.3733 to 0.4538)	1.734(0.9695 to 2.498)
BGC	1.039(0.9223 to 1.155)	0.7258(0.6675 to 0.7841)	2.426(1.624 to 3.228)
KATO	2.755(2.262 to 3.247)	0.4289(0.4031 to 0.4547)	3.718(2.742 to 4.694)
mkn45	3.198(2.536 to 3.860)	0.4323(0.3490 to 0.5155)	5.127(2.440 to 7.814)

IC_50_ values show Doxorubicin concentration [μg/ml. mean (95% confidence intervals)].

### The EMT underlies DOX resistance in GC cells

**Figure 3:**
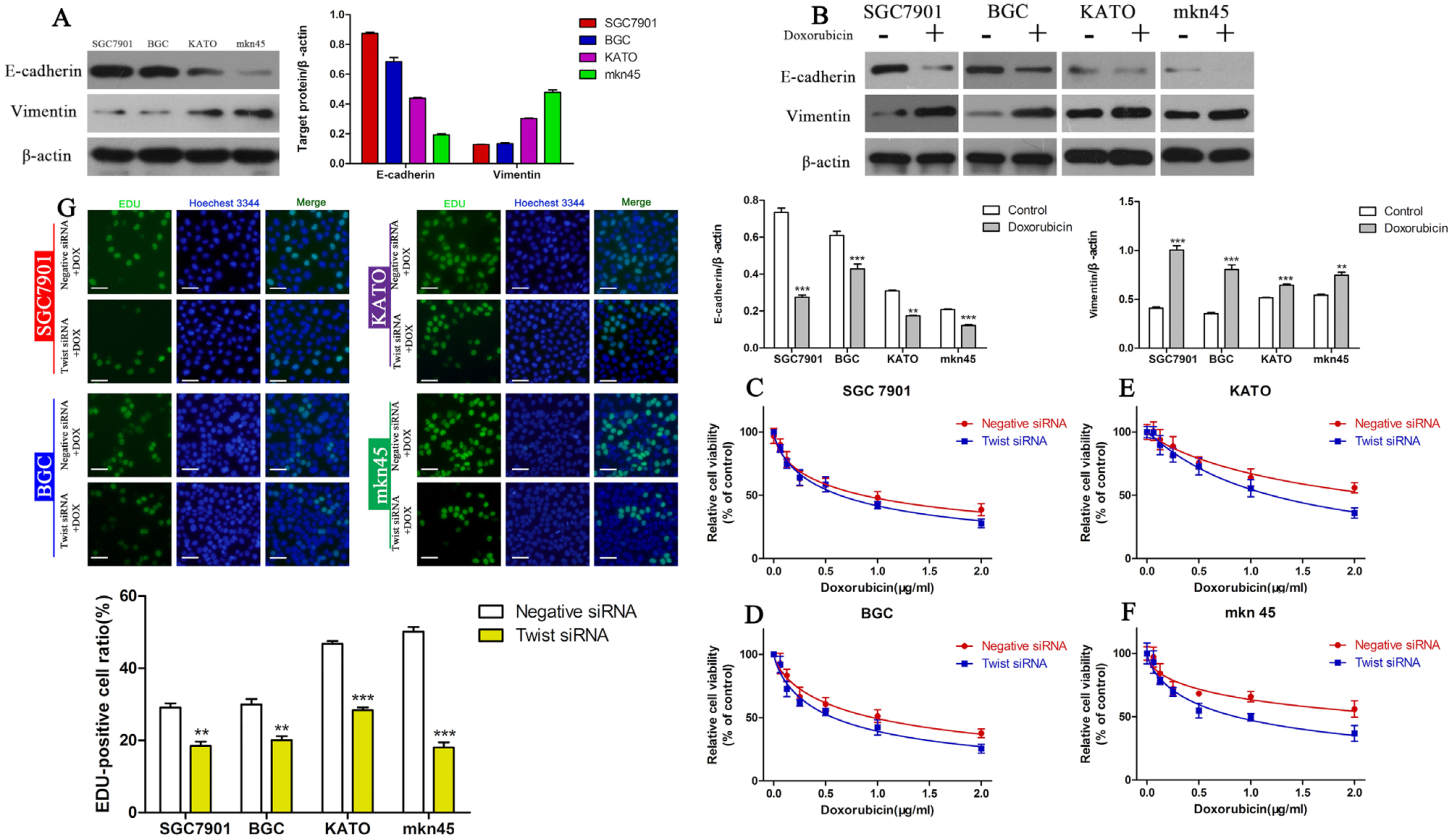
The EMT mediates DOX resistance in GC cell lines **(A)** Western blots of E-cadherin and vimentin protein expression in the four GC cell lines. **(B)** Western blots of E-cadherin and vimentin protein expression in the four GC cell lines cultured with their IC_50_ of DOX (^**^P < 0.01, ^***^P < 0.001). **(C-F)** CCK-8 assay of the viability of GC cells in which Twist was knocked down cultured in different concentrations of DOX. **(G)** EDU assay of the proliferation of GC cells in which Twist was knocked down cultured in their IC_50_ of DOX (^**^P < 0.01, ^***^P < 0.001).

As the EMT plays a role in chemoresistance in several solid tumors, we hypothesized the EMT may mediate DOX resistance in GC cells. To prove this hypothesis, we first investigated the expression of EMT marker proteins in the four GC cell lines by Western blotting. DOX-resistant DUSP4^high^ KATOIII and MKN45 cells expressed low levels of E-cadherin and high levels of vimentin, in contrast to DOX-sensitive DUSP4^low^ BGC and SGC7901 cells (Figure 3A). Treatment with the IC_50_ of DOX for 48 h increased vimentin expression and decreased E-cadherin expression compared to the respective untreated cells (Figure 3B) (^**^P < 0.01,^***^P < 0.001), indicating DOX can induce the EMT in GC cells.

Twist is an essential regulator of the EMT in cancer cells [31]. Therefore, we used a siRNA to knockdown Twist to inhibit the EMT. The CCK-8 assay revealed that knockdown of Twist increased the sensitivity of all four GC cell lines to DOX (Figure 3C-3F, Table 3) and the EDU assay confirmed these results (Figure 3G) (^**^P < 0.01,^***^P < 0.001), strongly suggesting that the EMT contributes to DOX resistance in GC cells.

**Table 3:** The viability of GC cells in which Twist was knocked down cultured in different concentrations of DOX

cell lines	IC_50_(μg/ml)	
	Negative siRNA+ DOX	Twist siRNA+ DOX
SGC7901	0.8609(0.7169 to 1.005)	0.6367(0.5718 to 0.7017)
BGC	0.9592(0.8089 to 1.110)	0.5751(0.5060 to 0.6443)
KATO	2.267(1.726 to 2.807)	1.195(1.057 to 1.334)
mkn45	2.765(1.650 to 3.879)	0.8573(0.7188 to 0.9957)

IC_50_ values show Doxorubicin concentration [μg/ml. mean (95% confidence intervals)].

**Figure 4:**
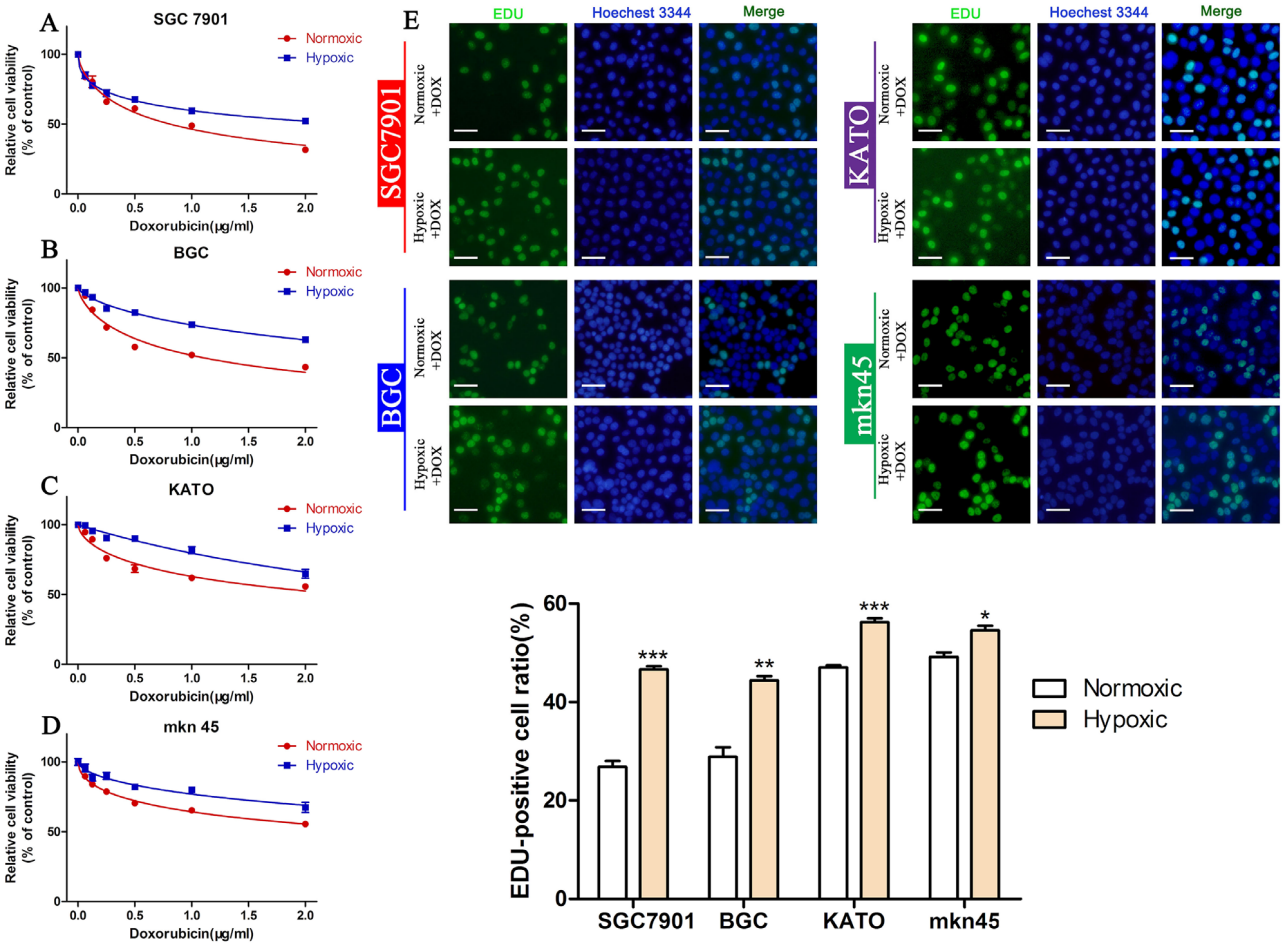
Hypoxia induces DOX resistance in GC cell lines **(A-D)** CCK-8 assay of the viability of GC cells treated with different concentrations of DOX cultured under hypoxic or normoxic conditions. **(E)** EDU assay of the proliferation of GC cells treated with different concentrations of DOX cultured under hypoxic or normoxic conditions (^*^P < 0.05, ^**^P < 0.01, ^***^P < 0.001).

Hypoxic culture can induce the EMT in cancer cell lines [32, 33]. To further confirm the role of the EMT in DOX resistance, we examined the viability and proliferation of GC cells cultured under hypoxic and normoxic conditions. Our results showed that DOX could reduce the viability of all four GC cell lines in a dose-dependent manner; however, cells cultured under hypoxic conditions were more resistant to DOX than those cultured under normoxic conditions (Figure 4A-4D, Table 4). The EDU assay confirmed hypoxia increased cell proliferation in a similar manner in DOX treated cells (Figure 4E) (^*^P < 0.05, ^**^P < 0.01,^***^P < 0.001). These findings prove the EMT promotes DOX resistance in GC cells.

**Table 4:** The viability of GC cells treated with different concentrations of DOX cultured under hypoxic or normoxic conditions

cell lines	IC_50_(μg/ml)	
	Normoxic+ DOX	Hypoxic+ DOX
SGC7901	0.8109(0.6978 to 0.9241)	2.429(1.813 to 3.045)
BGC	1.104(0.9518 to 1.256)	4.186(3.451 to 4.921)
KATO	2.365(1.759 to 2.971)	3.891(2.792 to 4.991)
mkn45	3.052(2.339 to 3.766)	7.746(2.586 to 12.91)

IC_50_ values show Doxorubicin concentration [μg/ml. mean (95% confidence intervals)].

### Knockdown of DUSP4 inhibits the EMT in GC cells

**Figure 5:**
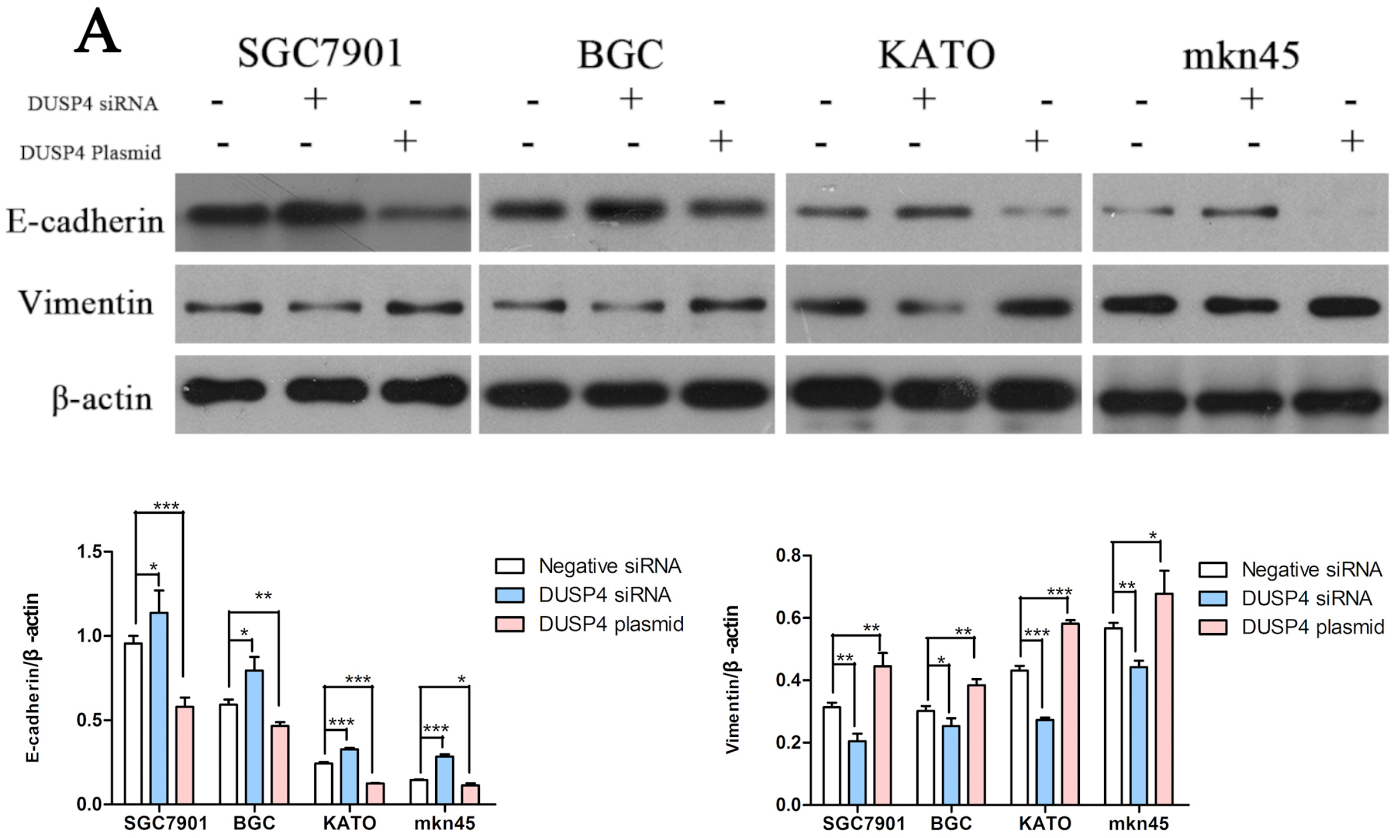
DUSP4 can induce the epithelial-mesenchymal transition (EMT) in GC cell lines Western blots of E-cadherin and vimentin protein expression in GC cells in which DUSP4 was knocked down or overexpressed (^*^P < 0.05, ^**^P < 0.01, ^***^P < 0.001).

Next, we investigated whether DUSP4 is involved in the EMT in GC cells. We transfected the *DUSP4* siRNA or DUSP4 plasmid into the four GC cell lines, and quantified the expression of E-cadherin and vimentin. Knockdown of *DUSP4* increased E-cadherin expression and reduced vimentin expression (Figure 5A). In contrast, overexpression of DUSP4 had the opposite effects (Figure 5A) (^*^P < 0.05, ^**^P < 0.01,^***^P < 0.001). These results indicated DUSP4 may regulate the EMT in GC cells.

### DUSP4 mediates DOX sensitivity in GC cells by regulating the EMT

**Figure 6:**
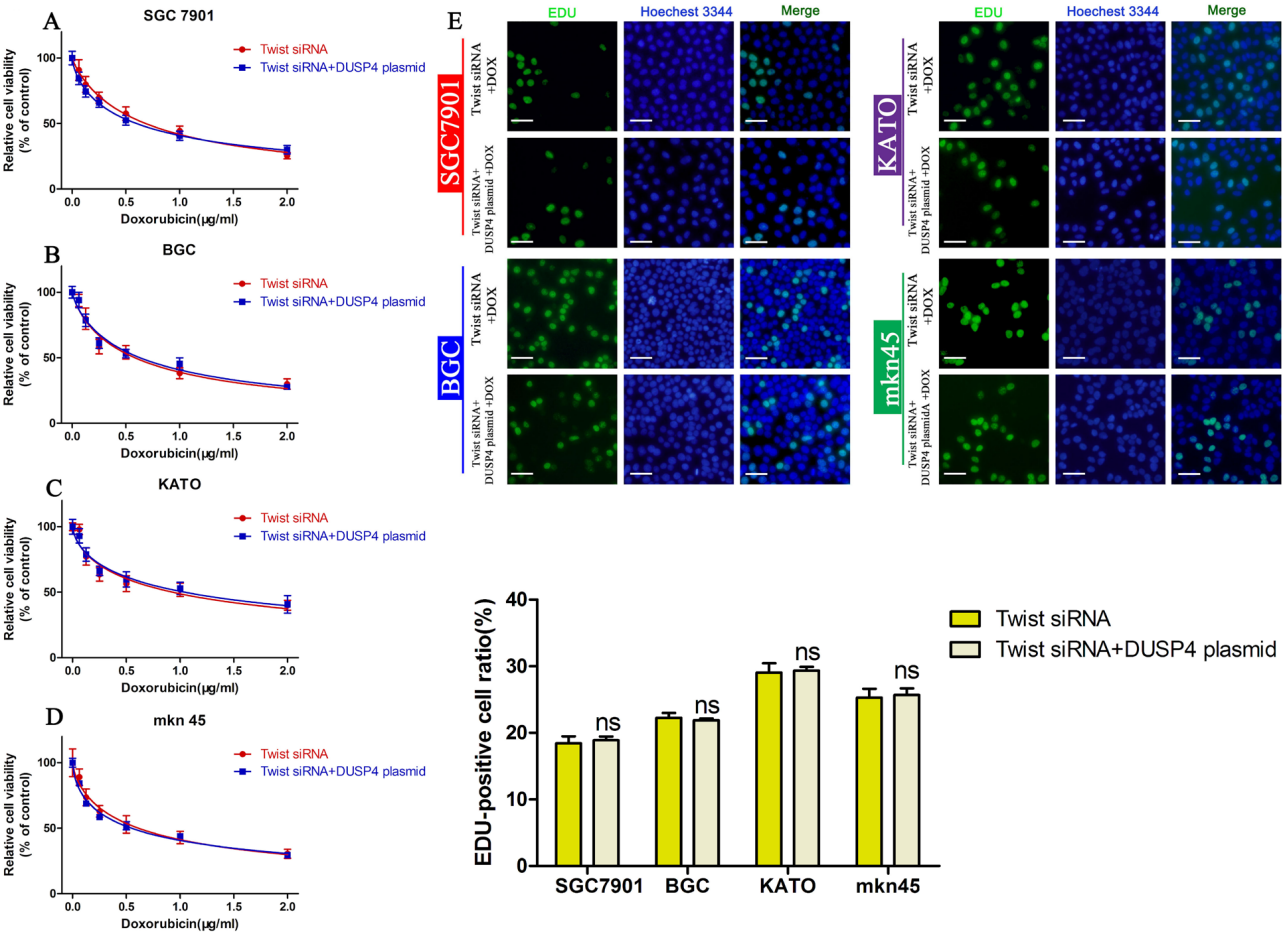
Blocking the EMT attenuates the ability of DUSP4 overexpression to induce DOX resistance **(A-D)** CCK-8 assay of the viability of GC cells transfected with the Twist siRNA or Twist siRNA + DUSP4 overexpressing plasmid treated with different concentrations of DOX. **(E)** EDU assay of the proliferation of GC cells transfected with the Twist siRNA or Twist siRNA + DUSP4 plasmid treated with their IC_50_ of DOX (NS: not significant).

Based on the results above, we inferred DUSP4 promotes DOX resistance in GC cells by regulating the EMT. To prove this, we examined the cell viability of cells overexpressing DUSP4, transfected with the Twist siRNA to inhibit the EMT and treated with DOX. Overexpression of DUSP4 did not further enhance DOX resistance when the EMT was blocked using the Twist siRNA (Figure 6A-6D, Table 5). The EDU assay confirmed these results (Figure 6E).

**Table 5:** The viability of GC cells transfected with the Twist siRNA or Twist siRNA + DUSP4 overexpressing plasmid treated with different concentrations of DOX

cell lines	IC_50_(μg/ml)	
	Twist siRNA+ DOX	TwistsiRNA+DUSP4 plasmid + DOX
SGC7901	0.6835(0.6131 to 0.7540)	0.5982(0.5448 to 0.6515)
BGC	0.5835(0.5047 to 0.6622)	0.6311(0.5534 to 0.7088)
KATO	0.9308(0.7426 to 1.119)	1.042(0.8521 to 1.233)
mkn45	0.6126(0.5211 to 0.7040)	0.5475(0.4976 to 0.5974)

values show Doxorubicin concentration [μg/ml. mean (95% confidence intervals)].

**Figure 7:**
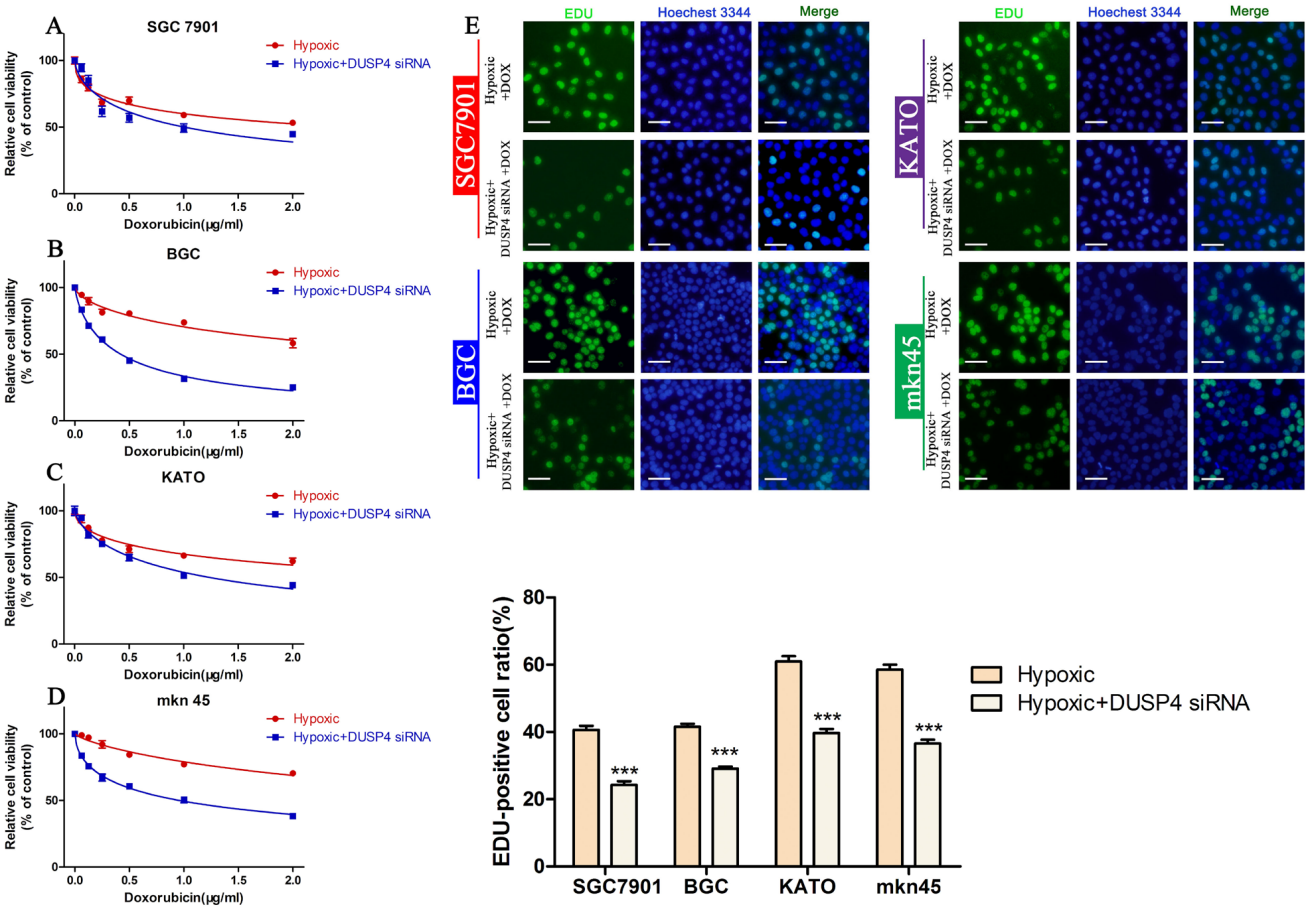
Knocking down *DUSP4* reverses hypoxia-induced DOX resistance **(A-D)** CCK-8 assay of the viability of GC cells exposed to hypoxia alone or hypoxia + *DUSP4* siRNA treated with different concentrations of DOX. **(E)** EDU assay of the proliferation of GC cells exposed to hypoxia alone or hypoxia + *DUSP4* siRNA treated with their IC_50_ of DOX (^***^P < 0.001).

As shown in Figure 4, hypoxia could induce the EMT and enhance DOX resistance in GC cells. We investigated whether knockdown of *DUSP4* could reverse the hypoxia-induced EMT and hypoxia-mediated DOX resistance. Cell viability and proliferation assays revealed that knockdown of *DUSP4* could alleviate hypoxia-mediated DOX resistance in GC cells (Figure 7A-7E, Table 6) (^***^P < 0.001). These data demonstrate that DUSP4 enhances DOX resistance by regulating the EMT in GC cells.

**Table 6:** The viability of GC cells exposed to hypoxia alone or hypoxia + DUSP4 siRNA treated with different concentrations of DOX

cell lines	IC_50_(μg/ml)	
	Hypoxic+DOX	Hypoxic+DUSP4 siRNA+DOX
SGC7901	2.503(1.544 to 3.463)	1.005(0.7426 to 1.268)
BGC	3.865(2.433 to 5.297)	0.4199(0.3964 to 0.4435)
KATO	4.156(2.366 to 5.946)	1.236(1.022 to 1.450)
mkn45	5.535(3.560 to 7.510)	0.9583(0.8292 to 1.087)

IC_50_ values show Doxorubicin concentration [μg/ml. mean (95% confidence intervals)].

## DISCUSSION

Chemotherapy is the most effective treatment for advanced GC [3]. However, drug resistance limits the application of conventional DOX-based chemotherapy regimens in GC [34]. Thus, it is essential to explore the mediators and mechanisms of drug resistance in GC. In this study, we showed that overexpression of DUSP4 enhanced DOX resistance in GC cell lines by regulating the EMT. To the best of our knowledge, this is the first report of a relationship between DUSP4 and DOX resistance in GC.

The dual specificity phosphatase DUSP4 specifically inactivates MAPK pathway kinases [18]. However, the role of DUSP4 in cancer remains controversial. Some studies have demonstrated DUSP4 is overexpressed and could promote progression in a number of cancers, including colorectal [19] and breast cancer [30, 35]. Conversely, other studies indicated DUSP4 is downregulated and inhibits tumor development in colorectal cancer [36–38] and breast cancer [25]. However, few studies have quantified DUSP4 expression in GC. One study reported that decreased DUSP4 expression was associated with sex, tumor size, depth of invasion and distant metastasis in human GC, and that overexpression of DUSP4 reduced GC cell viability and invasive potential and induced cell apoptosis and S phase cycle arrest [39]. However, little is known about the role of DUSP4 in chemoresistance in GC. The present study demonstrates DUSP4 promotes DOX resistance in GC, in agreement with findings in breast cancer [28].

The EMT confers increased invasion and migration ability and is associated with drug resistance [8, 9]. EMT-induced stemness may represent one potential mechanism underlying EMT-mediated chemoresistance [40]. A previous study showed that long-term, incremental DOX treatment led to drug resistance and the EMT in SGC7901/Dox cells [41]. Moreover, treatment with DOX for 48 h induced the EMT in human GC BGC-823 cells, and inhibition of β-catenin signaling suppressed the DOX-induced EMT and cell migration [16]. In agreement with these previous studies, we found the GC cell lines with a more mesenchymal phenotype, characterized by high vimentin and low E-cadherin expression, were more resistant to DOX. Furthermore, DOX induced vimentin and inhibited E-cadherin expression, indicting DOX induced the EMT in all four GC cell lines, and we confirmed the EMT mediates DOX resistance in GC.

Only two studies have reported a relationship between DUSP4 and the EMT. Boulding et al. demonstrated that DUSP1, DUSP4 and DUSP6 are induced during the EMT in breast cancer, and knockdown of *DUSP4* could enhance breast cancer stem celldefine (CSC) formation [42]. Liu Y et al. found that overexpression of DUSP4 may promote the EMT in breast cancer, whereas knockdown of *DUSP4* enhanced the sensitivity of chemotherapeutic agents in breast cancer cells [28]. Similarly, this study provides the first proof that DUSP4 can induce the EMT in GC and that blocking the EMT could reverse DUSP4-mediated DOX resistance in GC.

In conclusion, our data demonstrates that high DUSP4 expression may promote the EMT in GC, whereas low DUSP4 expression may enhance DOX sensitivity. However, further research is required to determine the mechanism(s) by which down-regulation of DUSP4 inhibits the EMT and decreases the chemosensitivity of GC cells. Furthermore, these findings indicate that DUSP4 may have potential as a biomarker for monitoring DOX resistance in patients with GC and could represent a novel treatment target for GC.

## MATERIALS AND METHODS

### Cell culture

BGC-823, KATO III, SGC7901 and MKN45 human gastric cancer cells were purchased from the American Type Culture Collection Cell Biology Collection (ATCC, Manassas, VA, USA). BGC-823, SGC7901 and MKN45 cells were maintained in RPMI-1640 (Gibco, Grand Island, NY, USA); KATO III cells, in DMEM-High glucose (Gibco); all culture media were supplemented with 10% fetal bovine serum (FBS) and cells were cultured at 37°C in a humidified atmosphere of 5% CO_2_. Hypoxic cultures were incubated at 37°C in a humidified atmosphere of 5% CO_2_ and 1% O_2_. Doxorubicin was obtained from Sigma (St. Louis, MO, USA) and dissolved in ddH2O.

### Cell transfection and RNA interference

GC cells were transfected with *DUSP4* siRNA (100 nmol/L; Santa Cruz Biotechnology, Santa Cruz, CA, USA) or Twist siRNA (100 nmol/L; Santa Cruz) using Lipofectamine 2000 (Invitrogen, Carlsbad, CA, USA) according to the manufacturer’s instructions. Non-specific negative control siRNA was purchased from GeneChem Co., Ltd. (Shanghai, China). Six hours after the cells were transfected, the media was replaced with fresh culture media. All assays were performed 48 h after transfection and repeated three times.

### DUSP4 overexpressing plasmid

The DUSP4 plasmid was designed and constructed by GeneChem Co., Ltd. using the Trans-OE expression vector. The DUSP4 plasmid or control vector (1 μg each) were transfected into GC cells using Lipofectamine 2000 (Invitrogen) according to the manufacturer’s instructions; cells were assayed at 48 h post-transfection.

### CCK-8 assay

Cell viability was assessed using the CCK-8 assay (Dojindo, Kumamoto, Japan). Approximately 5000 cells were seeded in 96-well plates in 100 μL medium. The cells were cultured in media containing 1% FBS for 24 h for synchronization, then different concentrations of DOX were added and the cells were incubated for 48 h. CCK-8 solution (10 μl) was added to each well, the cells were incubated for 2 h in the dark, then absorbance was measured at 450 nm using a MRX II microplate reader (Dynex, Chantilly, VA, USA). We used Prism 5 software (GraphPad, San Diego, CA, USA) to calculate the value of IC_50_.

### Western blotting

Cells were homogenized in Lysis Buffer (Cell Signaling Technology, Danvers, MA, USA), the supernatants were collected and total protein content was determined using the BCA assay (Thermo Fisher Scientific, Rockford, IL, USA). Equal amounts of protein (40 μg/lane) were separated on SDS-PAGE gels then transferred to 0.22 μm PVDF membranes (Millipore, Billerica, MA, USA). Membranes were blocked with 5% skimmed milk powder in PBST [0.1% Tween in PBS], incubated with E-cadherin, vimentin, β-actin or DUSP4 primary antibodies (1:1000; Abcam, Cambridge, MA, USA) overnight at 4°C, washed five times with PBST for 5 min, incubated with corresponding secondary antibodies (1:2000; abcam) for 1 h at room temperature, and target bands were visualized using the ECL kit (Millipore).

### EDU assay

Proliferation was determined using the Click-iTEdU Imaging Kit (Invitrogen) according to the manufacturer’s protocol. Briefly, cells were incubated with their IC_50_ of DOX for 24 h, fixed in 3.7% formaldehyde for 15 min at room temperature, and permeabilized in 0.5% Triton X-100 for 20 min at room temperature, washed twice with PBS containing 3% BSA, incubated with 0.5 mL of Click-iT^®^ reaction cocktail for 30 min in the dark, then nuclei were counterstained with 1 mL of 1 × Hoechst 33,342 (1:2000) for 30 min. The numbers of proliferative cells (EDU-positive) in three random fields of view per slide were counted under a fluorescence microscope (Olympus, Tokyo, Japan).

### Statistical analysis

SPSS17.0 software was used for statistical analysis. Data are expressed as the mean ± SD of three independent experiments and two groups were compared using the two-tailed Student’s *t*-test and one-way analysis of variance (ANOVA) was used to compare multiple groups. Statistical significance was accepted if *P* < 0.05.
